# Transcriptome dataset from *Solanum lycopersicum* L. cv. Micro-Tom; wild type and two mutants of INDOLE-ACETIC-ACID (SlIAA9) using long-reads sequencing oxford nanopore technologies

**DOI:** 10.1186/s13104-023-06306-1

**Published:** 2023-03-20

**Authors:** Wahyu Muhammad Yuha Lubis, M Adrian, Nurul Jadid, Ani Widiastuti, Hiroshi Ezura, Syariful Mubarok, Dhika Prita Hapsari, Roedhy Poerwanto, Deden Derajat Matra

**Affiliations:** 1 Study Program of Agronomy and Horticulture, Graduate School of IPB University, Bogor, Indonesia; 2 Department of Biology, Institut Teknologi Sepuluh Nopember, Surabaya, Indonesia; 3 Department of Plant Protection, Faculty of Agriculture, Universitas Gadjah Mada, Yogyakarta, Indonesia; 4 Faculty of Life and Environmental Sciences, University of Tsukuba, Tsukuba, Japan; 5 Department of Agronomy, Faculty of Agriculture, Universitas Padjadjaran, Sumedang, Indonesia; 6 Department of Agronomy and Horticulture, Faculty of Agriculture, IPB University, Bogor, Indonesia

**Keywords:** Climate change, Mutant, Parthenocarpy, RNA-seq

## Abstract

**Objective:**

Tomatoes are the most widely consumed fruit vegetable and are relatively easy to cultivate. However, an increase in temperature causes some plants to respond with a decrease in fruit production. So, it is necessary to develop plants resistant to extreme temperature changes. The tomato cv. Micro-Tom has genetic variations in the gene of INDOLE-ACETIC-ACID, namely SlIAA9-3 and SlIAA9-5. However, the genetic information regarding the full-length transcript of the gene from this type of tomato plant is unknown. Therefore, this study aimed to determine the full-length transcript of the genes of these three types of tomatoes using long-reads sequencing technology from Oxford Nanopore.

**Data description:**

The total RNA from three types of Micro-Tom was isolated with the RNeasy PowerPlant Kit. Then, the RNA sequencing process used PCR-cDNA Barcoding kit - SQK-PCB109 and continued with the processing of raw reads based on the protocol from microbepore protocol (https://github.com/felixgrunberger/microbepore). The resulting raw reads were 578 374, 409 905, and 851 948 for wildtype, iaa9-3, and iaa9-5, respectively. After obtaining cleaned reads, each sample was mapped to the tomato reference genome (*S. lycopersicum* ITAG4.0) with the Minimap2 program. In particular, 965 genes were expressed only in the iaa9-3 mutant, and 2332 genes were expressed only in the iaa9-5 mutant. Whereas in the wild type, 1536 genes are specifically expressed. In cluster analysis using the heatmap analysis, separate groups were obtained between the wild type and the two mutants. This proves an overall difference in transcript levels between the wild type and the mutants.

## Objective

Micro-Tom is a cultivar of a tomato that is affected by a mutation in the IAA9 gene, which IAA9 is a family member of Auxin/IAA (Indole-Acetic-Acid) transcription factors (T.F.) in tomato. The main role of this gene is formation in fruit-set. This antisense technology in the plant using AS-IAA9 shows several developmental defects, including strong parthenocarpy behavior related to IAA [1]. Some advantages of this mutant are higher production or yield in fruits, and it can survive under drought stress conditions [2].

Micro-Tom is small in size, has rapid growth and life cycle, easy transformation, and a short life cycle for fruit harvest [3], making Micro-Tom a convenient model for research in different fields. Several studies have been conducted on tomato genetics, like hormonal functions and interactions, carbohydrate metabolism, amino acids metabolism, and molecular breeding of tomato fruit shelf-life. The phenotype of Micro-Tom is due to at least three mutations, one of them is a dwarf (internode length reduction and smaller, rugose, dark-green leaves production) [4].

## Data description

A total of three RNA libraries (wild type, SlIAA9-3, SlIAA9-5) were prepared and sequenced (Data set 1, https://ddbj.nig.ac.jp/resource/sra-study/DRP009326). RNA-seq was performed using MinION ONT (Oxford Nanopore Technologies). Transcriptome sequencing had an estimated read of 578 374, 409 905, and 851 948 for wild type, SlIAA9-3, and SlIAA9-5, respectively. The results of sequencing and pre-processing are summarized in Data file 1 (Table 1, 10.6084/m9.figshare.21701345). After obtaining cleaned reads, each sample was mapped to the tomato reference genome (*S. lycopersicum* ITAG4.0) with the Minimap2 program. In particular, 965 genes were expressed only in the iaa9-3 mutant, and 2332 genes were expressed only in the iaa9-5 (Data file 2, 10.6084/m9.figshare.21701354). In cluster analysis using the heatmap method, separate groups were obtained between the wild type and the two mutants (Data file 3, 10.6084/m9.figshare.21701357).

The total RNA from young leaves was extracted using the RNeasy PowerPlant Kit (Qiagen) following the manufacturer’s protocol. The quality and quantity of RNA were checked by Nanophotometer NP-80 (Implen) and Qubit™ RNA Broad Range (B.R.) assay on Qubit® Fluorometer (Invitrogen). Then, the total RNA was subjected to RNA sequencing using PCR-cDNA Barcoding kit - SQK-PCB109 (PCB_9092_v109_revB_10Oct2019) [5]. The sequencing was performed on a Flow Cell R9.4.1 (FLO-MIN106D) on MinION Mk1B. After sequencing, the raw reads were base called using Guppy 6.1.2 with default parameters [6]. Next, data pre-processing followed https://github.com/felixgrunberger/microbepore protocol includes demultiplexing and NanoStat v1.2.1 to assess the reads quality and reads’ statistics [5, 7]. Next, full-length reads with remaining SSP (strand-switching primer) and VNP (oligo-dT30VN) primers were identified using pychopper v2.5.0 (https://github.com/nanoporetech/pychopper). Then, polyA-tails and the remaining SSP adapters were removed using Cutadapt [8]. The cleaned reads were mapped to the public tomato reference genome (*S. lycopersicum* ITAG4.0) using Minimap2 [6, 9]. To estimate gene abundance in each sample, the mapped-clean reads were calculated in alignment-based mode using salmon v1.9.0 [10]. Finally, transcripts per million (TPM) from each treatment were compared using clustering analysis by using RStudio 4.1.2 version [11] with some packages; gplots, cluster, and heatmap2.

**Table 1:** Overview of data files/data sets

Label	Name of data file/data set	File types (file extension)	Data repository and identifier (DOI or accession number)
Data set 1	Raw RNA-seq reads	Fastq files (.fastq)	https://ddbj.nig.ac.jp/resource/sra-study/DRP009326 [12]
Data file 1	Summary of raw and clean reads and transcriptome assembly	Document file (.docx)	10.6084/m9.figshare.21701345 [13]
Data file 2	Venn diagram for comparison of the number of expressed genes in wild type, iaa9-3 and iaa9-5 mutants	png file (.png)	10.6084/m9.figshare.21701354 [14]
Data file 3	Clustering analysis of gene abundance estimation using Heatmaps based on de novo assembled transcript	png file (.png)	10.6084/m9.figshare.21701357 [15]

## Limitations

This study had limitations in obtaining a good-quality total RNA without degradation and fragmentation during library construction. In addition, the heat stress treatment which stresses makes the plant difficult to survive in high heat conditions.

## Data Availability

Raw FASTQ files were deposited in the DDBJ database under accession number DRP009326 https://ddbj.nig.ac.jp/resource/sra-study/DRP009326 [9].

## Abbreviations

RNA: Ribonucleic acid
RNA-seq: RNA sequencing
